# Rhes depletion promotes striatal accumulation and aggregation of mutant huntingtin in a presymptomatic HD mouse model

**DOI:** 10.3389/fnagi.2023.1237018

**Published:** 2023-08-10

**Authors:** Yongcheng Pan, Beisha Tang, Xiao-Jiang Li, Shihua Li, Qiong Liu

**Affiliations:** ^1^Department of Neurology, Xiangya Hospital, Central South University, Changsha, Hunan, China; ^2^Key Laboratory of Hunan Province in Neurodegenerative Disorders, Xiangya Hospital, Central South University, Changsha, Hunan, China; ^3^National Clinical Research Center for Geriatric Disorders, Xiangya Hospital, Central South University, Changsha, Hunan, China; ^4^Guangdong Key Laboratory of Non-human Primate Research, Key Laboratory of CNS Regeneration (Ministry of Education), GHM Institute of CNS Regeneration, Jinan University, Guangzhou, China

**Keywords:** Rhes, mHTT aggregates, neurodegeneration, Huntington’s disease, gene targeting

## Abstract

**Introduction:**

Huntington’s disease (HD) is caused by CAG trinucleotide repeats in the HTT gene. Selective neurodegeneration in the striatum is prominent in HD, despite widespread expression of mutant HTT (mHTT). Ras homolog enriched in the striatum (Rhes) is a GTP-binding protein enriched in the striatum, involved in dopamine-related behaviors and autophagy regulation. Growing evidence suggests Rhes plays a critical role in the selective striatal degeneration in HD, but its specific function in this context remains complex and controversial.

**Methods:**

In this study, we utilized CRISPR/Cas9 to knockdown Rhes at different disease stages through adeno-associated virus (AAV) transduction in HD knock-in (KI) mice. Immunoblotting and immunofluorescence were employed to assess the impact of Rhes depletion on mHTT levels, neuronal loss, astrogliosis and autophagy activity.

**Results:**

Rhes depletion in 22-week-old HD KI mice (representing the presymptomatic stage) led to mHTT accumulation, reduced neuronal cell staining, and increased astrogliosis. However, no such effects were observed in 36-week-old HD KI mice (representing the symptomatic stage). Additionally, Rhes deletion in 22-week-old HD KI mice resulted in increased P62 levels, reduced LC3-II levels, and unchanged phosphorylation of mTOR and beclin-1, unchanged mTOR protein level, except for a decrease in beclin-1.

**Discussion:**

Our findings suggest that knockdown Rhes promotes striatal aggregation of mutant huntingtin by reducing autophagy activity in a mTOR-independent manner. Rhes plays a protective role during the presymptomatic stage of HD KI mice.

## Introduction

Huntington’s disease (HD) is a neurodegenerative disorder caused by the expansion of CAG trinucleotide repeats in the *Huntingtin* (HTT) gene (The Huntington’s Disease Collaborative Research Group, 1993; Ross and Tabrizi, 2011). In HD, the most prominent pathological change is the selective neurodegeneration in the striatum despite the ubiquitous expression of mHtt throughout the brain and body (Vonsattel and DiFiglia, 1998; Walker, 2007). Although the progressive accumulation of mutant HTT (mHTT) is known to contribute to selective neurodegeneration in the striatum (Vonsattel et al., 1985; Bates et al., 2015), the mechanism underlying this process remains to be investigated.

Rhes, also known as Ras homolog enriched in the striatum, is a GTP-binding protein that is encoded by the *Rasd2* gene (Spano et al., 2004). It is involved in mediating dopamine-related behaviors and signaling pathways, serving as an inhibitor of motor stimuli in the striatum (Falk et al., 1999; Vargiu et al., 2001; Shahani et al., 2016; Napolitano et al., 2018). Additionally, Rhes plays a crucial role in regulating autophagy. It has been reported that Rhes may regulate the activity of mammalian target of rapamycin (mTOR) (Subramaniam et al., 2011), a key signaling pathway involved in cell growth, metabolism, and autophagy. Moreover, Rhes has been suggested to induce autophagy through mTOR-independent pathways (Mealer et al., 2014), potentially impacting protein quality control and clearance.

Increasing evidence supports the pathogenic role of Rhes in the selective striatal degeneration observed in HD. In HD cell models, Rhes promotes the accumulation of mHTT protein and mediates its cytotoxicity (Subramaniam et al., 2009). Knockdown of Rhes is protective in the htt171-82Q primary striatal neuron model of HD (Seredenina et al., 2011). Rhes knockout mice were protected from neurotoxicity and motor dysfunction in a HD model elicited by 3-nitropropionic acid (Mealer et al., 2013). It is reported that genetic knockout of Rhes in the R6/1 HD mice significantly delayed the onset of HD-like symptoms like motor dysfunction and dystonia (Baiamonte et al., 2013). Moreover, Rhes deletion prevents striatal atrophy, brain weight loss, and psychiatric-like behavioral deficits in N171-82Q mice (Swarnkar et al., 2015). Conversely, ectopic overexpression of Rhes in the cerebellum of N171-82Q HD transgenic mice accelerates motor deficits and neurodegeneration (Swarnkar et al., 2015).

However, conflicting data have been reported regarding the role of Rhes in HD. On one hand, Rhes levels are reduced in the brains of HD patients (Hodges et al., 2006). A study showed that genetic knockout of Rhes in wild-type mice affects body weight, motor coordination, and psychiatric behaviors (Spano et al., 2004; Heikkinen et al., 2021). Moreover, Rhes suppression led to increased anxiety-like behaviors and enhanced striatal atrophy in BACHD mice (Lee et al., 2014). On the other hand, overexpression of Rhes alleviates motor deficits and improves brain pathology in N171-82Q mice (Lee et al., 2015). Intriguingly, a recent study reported that genetic Rhes knockout does not substantially ameliorate or exacerbate HD phenotypes in HD Q175 mice (Heikkinen et al., 2021). Given these discrepancies, further *in vivo* investigations are required to determine the nuanced role of Rhes in HD.

In this study, we aimed to investigate the effects of Rhes knockdown using CRISPR/Cas9 at different disease stages in HD knock-in (KI) mice. Through the use of adeno-associated virus (AAV) transduction, we observed that the depletion of Rhes in HD KI mice resulted in the accumulation of mHTT, decreased neuronal cell staining, and increased astrogliosis specifically in the 22-week-old (representing the presymptomatic stage) HD KI mice. However, no such effects were observed in the 36-week-old (representing the symptomatic stage) HD KI mice. Considering the involvement of Rhes in regulating autophagy, an essential process that clears misfolded protein, we examined the level of P62 and LC3-II, two known markers of autophagy activity. We found an increase in P62 levels and a reduction of LC3-II levels upon Rhes deletion in 22-week-old HD KI mice. Furthermore, Western blotting results revealed that the protein level of mTOR, phosphorylation of mTOR, and phosphorylation of beclin-1 remained unchanged, while beclin-1 was decreased. These results suggested that Rhes deletion may lead to a reduction in autophagy activity through an mTOR-independent manner. Our studies suggest a potential protective role of Rhes during the presymptomatic stage of HD KI mice.

## Results

### Efficient knockdown of Rhes via CRISPR/Cas9 both *in vitro* and *in vivo*

**FIGURE 1:**
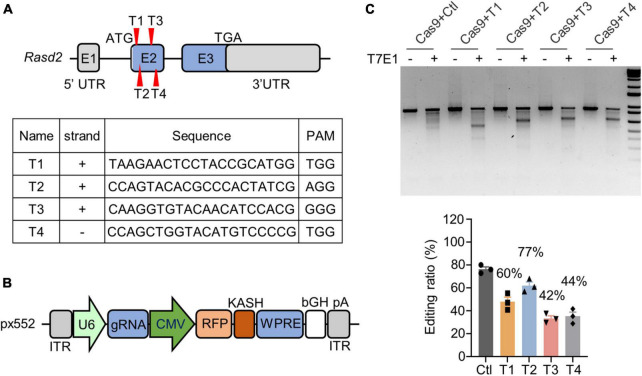
Knockdown of Rhes via CRISPR/Cas9 *in vitro*. **(A)** Schematics and sequences of the designed *Rasd2* guide RNAs. Rhes in mouse is encoded by R*asd2* gene (NM_029182), which has three exons. Four guide RNAs (T1–T4) were designed to target exon2 of the *Rasd2* gene. **(B)** Schematic diagram of AAV-*Rasd2* gRNA vectors. HA, human influenza hemagglutinin; ITR, inverted terminal repeat; KASH, Klarsicht; WPRE, woodchuck hepatitis virus post-transcriptional regulatory element. **(C)** T7E1 assays verified that CRISPR/Cas9 targeting could cause mutations in the *Rasd2* gene (top) and the editing ratio was analyzed (bottom). Three independent experiments were performed.

Ras homolog enriched in striatum, which is encoded by the *Rasd2* gene, was targeted for knockdown via CRISPR/Cas9 technology by using four guide RNAs (gRNAs) designed to target exon 2 of the mouse *Rasd2* gene (T1, T2, T3, and T4) (Figure 1A). These gRNAs were then subcloned into a px552 vector, which expressed the gRNAs under the U6 promoter and red fluorescent protein (RFP) under the CMV promoter (Figure 1B). The expression of RFP served as an indicator of *Rasd2* gRNA expression. To assess the targeting efficiency of *Rasd2* gRNAs, we transfected them along with a Cas9 plasmid into cultured mouse neuroblastoma cells (N2a). Analysis of the DNA using T7E1 digestion revealed that *Rasd2* gRNAs efficiently edited the *Rasd2* gene (Figure 1C). For further validation *in vivo*, we selected two gRNAs (T3 and T4) that exhibited higher editing efficiency in N2a cells.

**FIGURE 2:**
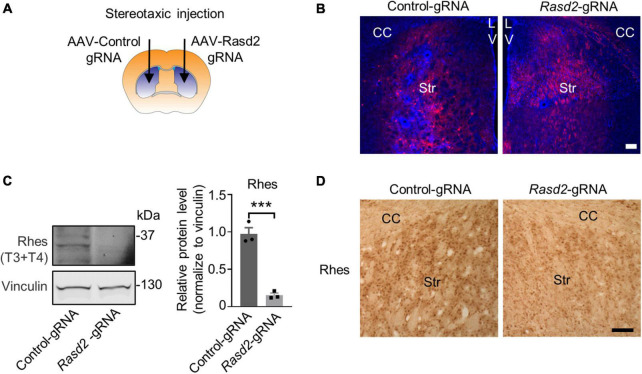
Knockdown of Rhes in mouse striatum via AAV transduction. **(A)** Schematic diagram of bilateral stereotaxic injection of the striatum in mice. One side of the striatum of Cas9 transgenic mice was injected with AAV-*Rasd2*-gRNAs and the contralateral side was injected with AAV-control-gRNA. Two microliter of AAV-gRNA virus (10^12^ genome copies/ml) per side. **(B)** Representative images showed the expression of AAV-control gRNA and AAV-*Rasd2* gRNAs in the striatum. CC, corpus callosum; Str, striatum; LV, lateral ventricles. Blue, DAPI; red, RFP, from the AAV vector. **(C)** Western blotting verified that the protein level of Rhes was markedly reduced in the AAV-*Rasd2*-gRNAs injected side, compared to the contralateral side injected with AAV-control-gRNA. The data are presented as mean ± SEM; Student’s *t*-test, ***P = 0.0007, *n* = 3 per group. **(D)** Immunostaining of Rhes in the AAV-control gRNA and AAV-*Rasd2* gRNA-injected striatum. *N* = 4 per group. Scale bars, 100 μm.

To assess the targeting effect of *Rasd2* gRNAs *in vivo*, we packaged the px552 gRNA into an AAV9 virus. Stereotaxic injection of AAV viruses expressing control gRNA or *Rasd2* gRNA was performed in the striatum of Cas9 mice (Figure 2A), that express Cas9 ubiquitously under the CAG promoter (Liu et al., 2020a; Yang et al., 2020). AAV-*Rasd2*-gRNAs of T3 and T4, either alone or in combination, were injected into one side of the striatum of 2-month-old Cas9 mice. The contralateral side was injected with AAV-control-gRNA, which did not target any known gene (Yang et al., 2017), to rule out non-specific effects caused by viral transduction. After 28 days, the brains of the AAV-injected mice were dissected, and the distribution of RFP fluorescence signal confirmed the predominant expression of *Rasd2* gRNA in the striatum (Figure 2B). Western blotting analysis revealed a significant reduction in Rhes levels on the side injected with AAV-*Rasd2* gRNAs (mixture of T3 and T4) compared to the contralateral side injected with AAV-control-gRNA (Figure 2C). This finding was further validated by immunohistochemical staining against Rhes in the AAV-injected brains (Figure 2D), confirming the efficient elimination of Rhes *in vivo* through AAV-*Rasd2*-gRNAs injection.

### Knockdown of Rhes accelerates the aggregation of mHTT at the presymptomatic stage of HD KI mice

**FIGURE 3:**
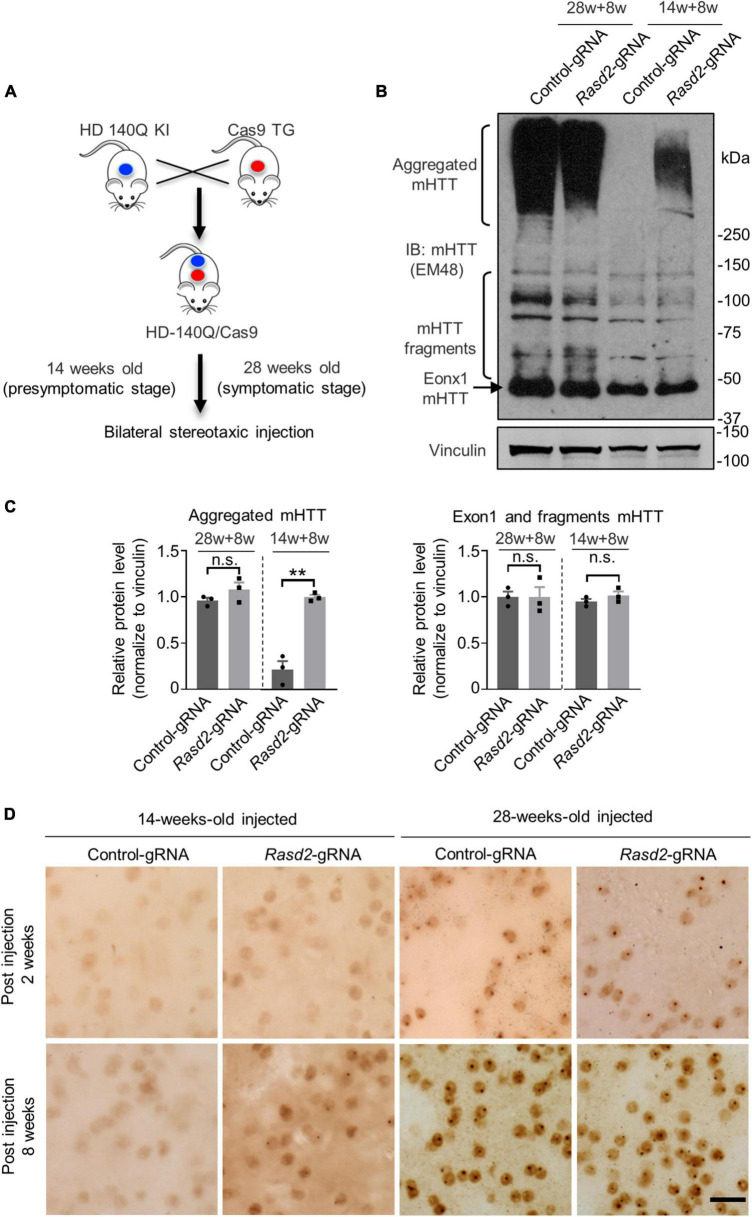
Knockdown of Rhes increases mHTT accumulation during the presymptomatic stage of HD KI mice. **(A)** HD-140Q/Cas9 (referred to as HD KI) mice were generated by crossing HD-140Q KI mice with Cas9 transgenic mice. AAV-control gRNA and AAV-*Rasd2* gRNA were respectively injected into the contralateral sides of the striatum of HD KI mice as indicated in Figure 2A at 14 weeks old or 28 weeks old of age. Eight weeks after injection (14 + 8 weeks, representing the presymptomatic stage, or 28 + 8 weeks, representing the symptomatic stage), injected brains were examined. **(B)** Western blotting analysis of mHTT in the AAV-control gRNA and AAV-*Rasd2* gRNA-injected striatum at different disease stages. Vinculin served as a loading control. **(C)** Densitometric ratios of aggregated mHTT (left panel) and exon1 and fragments mHTT (right panel) to vinculin on western blots in panel **(B)**. The data are presented as mean ± SEM; Student’s *t*-test, **P = 0.0011, ^n^.^s^.not significant, *n* = 3 per group. **(D)** Immunostaining of mHTT in the AAV-control gRNA and AAV-*Rasd2* gRNA-injected striatum at different disease stages. *N* = 4 per group. Scale bars, 20 μm.

Previous studies have indicated that genetic knockout of Rhes during the embryonic stage can lead to abnormal physiological function in mice at a certain age (Spano et al., 2004; Heikkinen et al., 2021). Considering this, we decided to knockdown Rhes at different disease stages in the HD mouse model. The HD 140Q/Cas9 (referred to as HD KI) mice, which were generated previously by crossing HD 140Q KI mice with Cas9 transgenic mice (Liu et al., 2020a; Yang et al., 2020), were used in our study (Figure 3A). These mice exhibit intranuclear mHTT aggregations in the striatum and behavioral defects starting at 7 months old (28 weeks old), similar to the HD140Q KI mice (Yang et al., 2020). AAV injection was performed at 14 weeks old and 28 weeks old, corresponding to the presymptomatic and symptomatic stages, respectively (Figure 3A). One side of the HD KI mouse striatum was injected with AAV-*Rasd2* gRNA, while the contralateral striatum was injected with AAV-control gRNA. Eight weeks after injection, the injected brains were examined (Figure 3A).

Since the striatum is the most affected brain region in HD and Rhes are mainly expressed in the striatum, we focused on the effect of Rhes knockdown on the striatum. To assess the impact of Rhes knockdown on the level of mHTT in the striatum, we examined the levels of mHTT in the injected mice. Intriguingly, in the 14-weeks-old-injected group, Western blotting showed a significant increase in the amount of mHTT aggregates in the AAV-*Rasd2* gRNA injected side of the striatum after 8 weeks of injection, while no mHTT aggregation was observed in the AAV-control gRNA injected side (Figures 3B, C). However, in the 28-weeks-old-injected group, a substantial amount of aggregated mHTT was found on both sides of the striatum, with no significant difference (Figures 3B, C). In addition, no significant alternations were found on the level of fragments mHTT including exon1 mHTT, which were proteolyzed from full-length mHTT and play key roles in the pathogenesis of HD (Landles et al., 2010; Yang et al., 2020) (Figures 3B, C). To further validate these findings, the injected brain slices were subjected to immunohistochemistry using mEM48 staining. The results confirmed that AAV-*Rasd2* gRNA increased the amount of aggregated mHTT in the 14-weeks-old-injected group (Figure 3D). These results indicate that knockdown of Rhes accelerates the aggregation of mHTT at the presymptomatic stage of HD KI mice.

### Knockdown of Rhes causes NeuN-positive cell reduction in the striatum of HD KI mice at the presymptomatic stage

**FIGURE 4:**
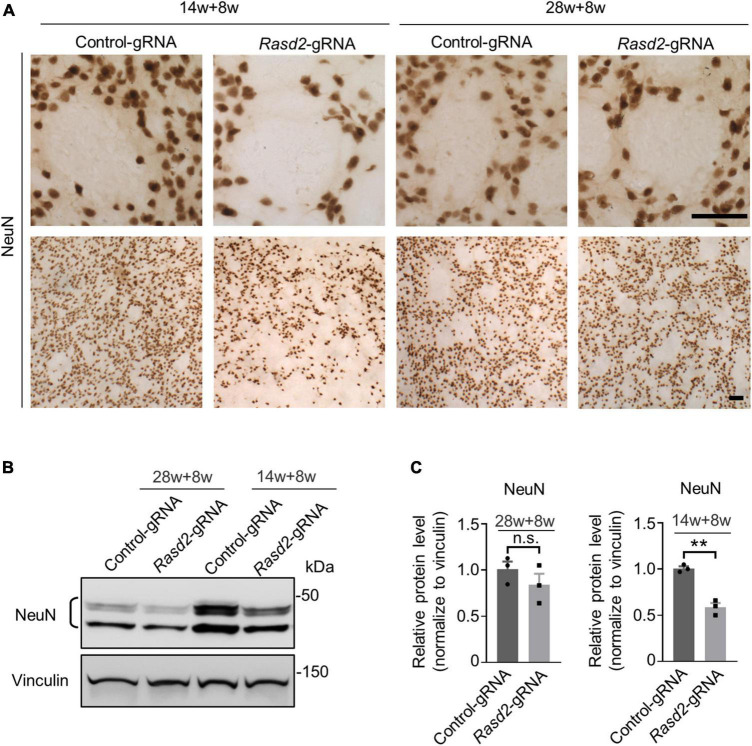
Knockdown of Rhes causes decreased NeuN in the striatum during the presymptomatic stage of HD KI mice. **(A)** Immunostaining of NeuN in the AAV-control gRNA and AAV-Rasd2 gRNA-injected striatum at different disease stages. *N* = 4 per group. Scale bars, 50 μm. **(B)** Western blotting showed the reduced expression of NeuN in the AAV-*Rasd2* gRNA-injected striatum at the age of 14 + 8 weeks, compared to the AAV-control-gRNA-injected side. **(C)** Densitometric ratios of NeuN to the vinculin were presented. The data are presented as mean ± SEM, Student’s *t*-test, **P = 0.0013, *n* = 3 per group.

Since preferential neuronal loss in the striatum is an important feature in HD patient brains, we aimed to investigate the impact of Rhes knockdown on neuronal loss in HD KI mice. Immunohistochemistry results revealed a notable reduction in NeuN staining intensity in the striatum of the AAV-*Rasd2* gRNA-injected side as compared to the contralateral AAV-control gRNA-injected side in the 14-weeks-old-injected group (Figure 4A). However, in the 28-weeks-old-injected group, the difference in NeuN staining between the AAV-Rasd2 gRNA-injected side and the contralateral AAV-control gRNA-injected side was negligible (Figure 4A). Western blotting confirmed a significant decrease in NeuN levels on the AAV-Rasd2 gRNA-injected side compared to AAV-control gRNA-injected side in the 14-weeks-old injected group (Figures 4B, C).

**FIGURE 5:**
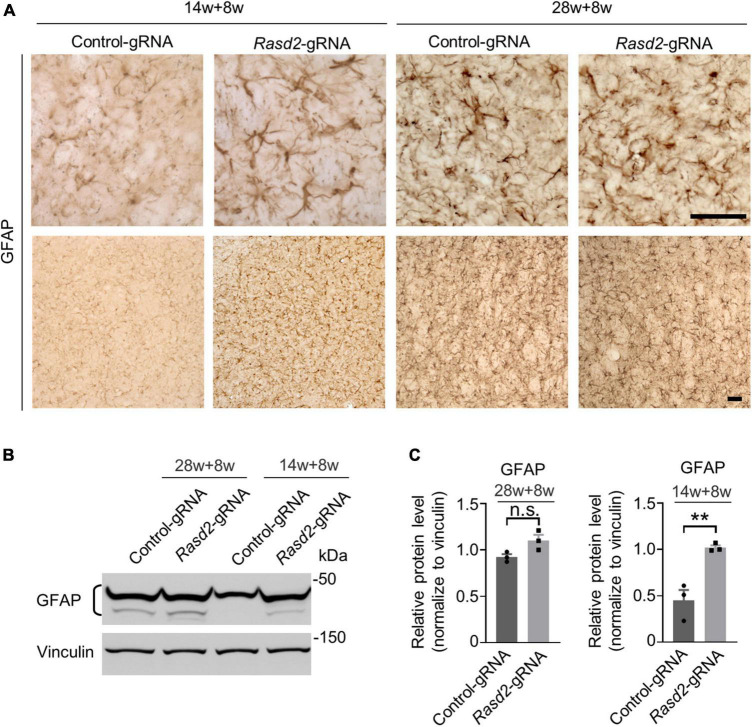
Knockdown of Rhes causes astrogliosis in the striatum during the presymptomatic stage of HD KI mice. **(A)** Immunostaining of GFAP in the AAV-control gRNA and AAV-*Rasd2* gRNA-injected striatum at different disease stages. *N* = 4 per group. **(B)** Western blotting showed the increased expression of GFAP in the AAV-*Rasd2* gRNA-injected striatum at the age of 14 + 8 weeks, compared to the AAV-control-gRNA-injected side. **(C)** Densitometric ratios of GFAP to the vinculin were presented. The data are presented as mean ± SEM, Student’s *t*-test, **P = 0.0081, *n* = 3 per group. Scale bars, 50 μm.

Next, we examined astrogliosis, another hallmark of neurodegeneration characterized by reactive astrocytes with increased GFAP staining (Yang et al., 2017). As expected, GFAP staining was significantly elevated in the striatum of HD KI mice during the symptomatic stage. However, knockdown of Rhes did not notably increase GFAP staining in the 28-weeks-old-injected group (Figure 5A). Conversely, in the 14-weeks-old injected HD KI mice, GFAP staining was significantly increased in the AAV-*Rasd2* gRNA-injected side compared to the contralateral AAV-control gRNA-injected side (Figures 5B, C).

### Knockdown of Rhes leads to reduced autophagy activity during the presymptomatic stage

Ras homolog enriched in striatum has been previously implicated as a modulator of autophagy (Mealer et al., 2014), a vital process involved in the clearance of misfolded proteins. Our results suggested that the depletion of Rhes led to the accumulation of mHTT in the striatum of HD KI mice at 22 weeks old (8 weeks after injection), suggesting a potential disturbance in autophagy during the presymptomatic stage.

**FIGURE 6:**
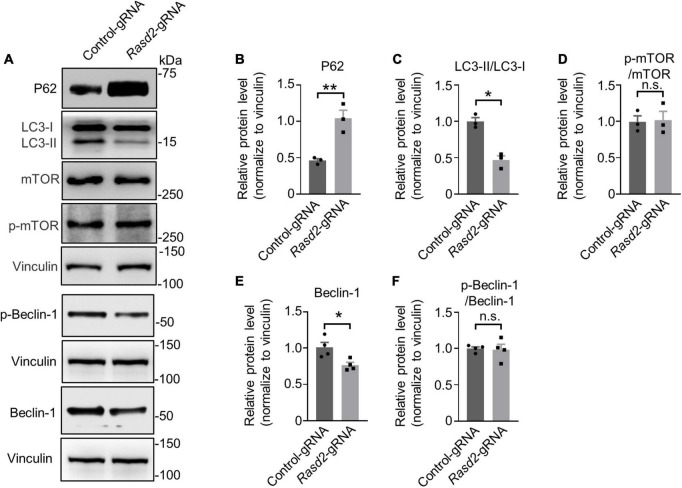
Knockdown of Rhes leads to reduced autophagy activity in the striatum during the presymptomatic stage of HD KI mice. **(A)** Western blotting analysis of P62, LC3, Phospho-mTOR (p-mTOR), mTOR, p-Beclin-1, and Beclin-1 in the AAV-control gRNA and AAV-*Rasd2* gRNA-injected striatum at the age of 14+ 8 weeks, compared to the AAV-control-gRNA-injected side. Densitometric ratios of P62 **(B)**, LC3-II/LC3-I **(C)**, p-mTOR/mTOR **(D)**, Beclin-1 **(E)**, and p-Beclin-1/Beclin-1 **(F)** to the vinculin were presented. The data are presented as mean ± SEM, Student’s *t*-test, **P = 0.0074 (P62), *P = 0.0028 (LC3-II/LC3-I), *P = 0.0223 (Beclin-1), *n* = at least 3 per group.

To investigate the impact of Rhes knockdown on autophagy, we first examined the levels of P62, an autophagy substrate that is used as a reporter of autophagy activity (Johansen and Lamark, 2011). Western blotting analysis of the *Rasd2* gRNA-injected and control-gRNA-injected striatum showed a significant increase in P62 levels in the AAV-*Rasd2* gRNA-injected side, while no significant change was observed on the contralateral AAV-control gRNA-injected side (Figures 6A, B). For further investigation, we check the status of LC3, the most widely used autophagosome marker. Western blotting results showed a decrease of LC3-II and LC3-II/LC3-I in the AAV-*Rasd2* gRNA-injected side (Figures 6A, C). Increased P62 level and decreased LC3-II suggested reduced autophagy activity during the presymptomatic stage upon Rhes knockdown.

Previous reports have indicated that Rhes may bind and activate mTOR to inhibit autophagy (Subramaniam et al., 2011). Nevertheless, we did not observe changes in mTOR activity, indicated by unaltered levels of mTOR and phosphorylation of mTOR (Figures 6A, D), after Rhes deletion. Considering Rhes’s potential role in regulating autophagy through an mTOR-independent mechanism involving Beclin-1 (Kang et al., 2011; Mealer et al., 2014; Menon and Dhamija, 2018), we assessed the protein level of Beclin-1 and its phosphorylation. A decreased protein level of Beclin-1 (Figures 6A, E, F) was found in the AAV-*Rasd2* gRNA-injected side, which further supports impaired autophagy during the presymptomatic stage. These results suggested that Rhes deletion may lead to decreased autophagy activity through an mTOR-independent manner.

## Discussion

Modulating the protein level of Rhes has been proposed as a potential therapeutic approach for HD. However, conflicting findings regarding the role of Rhes in HD have been reported on multiple occasions (Subramaniam et al., 2009; Baiamonte et al., 2013; Lee et al., 2014, 2015; Swarnkar et al., 2015) using cell models or transgenic HD mouse models. Given that mHTT is the prime culprit for HD and its protein level influences the disease progression (Yang et al., 2017; Li et al., 2019), we investigated the effect of Rhes deletion on mHTT level and neuropathology in the striatum at different disease stages in the HD KI mouse model. Our results showed that deletion of Rhes at an early age of the HD KI mice, resulted in increased mHTT aggregates, while at a later stage, there is no significant change of mHTT level or aggregates.

It has been demonstrated that autophagy is the primary clearance system for aggregate-prone proteins such as mHTT, and Rhes may play a role in regulating autophagy activity (Ravikumar et al., 2002; Williams et al., 2008; Mealer et al., 2014; Li et al., 2019). Therefore, we hypothesized that the depletion of Rhes could also impact autophagy. When Rhes was knockdown in HD KI mice at 22 weeks old, there was a decrease in LC3-II level and an increase in P62 level. The increase in p62 levels is often observed when autophagosome synthesis or maturation or autophagic degradation is inhibited or compromised, while the decrease in LC3-II may indicate either a decrease in autophagosome synthesis or an enhancement of autophagic degradation (Mizushima and Yoshimori, 2007; Johansen and Lamark, 2011; Yoshii and Mizushima, 2017). These results suggest that reduced or impaired autophagy activity may result from decreased autophagosome synthesis. Previous studies have reported that Rhes can modulate autophagy in both mTOR-dependent or independent manners (Kang et al., 2011; Subramaniam et al., 2011; Mealer et al., 2014; Menon and Dhamija, 2018). Nevertheless, we did not see changes in mTOR activity after Rhes deletion, which aligns with a previous study (Heikkinen et al., 2021). The effect of Rhes deletion on mTOR activity may be compensated by Rheb (Ras homolog enriched in brain) (Long et al., 2005). It has been reported that the release of Beclin-1 from Bcl2/Bcl-XL proteins seems to be an essential step for initiating the autophagic response (Kang et al., 2011; Menon and Dhamija, 2018) and Rhes may bind Beclin-1 and competitively displace Bcl2 from Beclin-1 to induce autophagosome synthesis (Mealer et al., 2014), it is plausible that Rhes deletion may disturb autophagy induction. In addition, reduced Beclin-1 may also cause autophagy deficiency (Yue et al., 2003; Salminen et al., 2013). Our Western blotting showed that Beclin-1 was decreased after knocking down of Rhes, although the one phosphor-Beclin-1 (p-Beclin-1) antibody did not show a change of p-Beclin-1 (ser15), which might not be the correct phosphorylation site. These results suggested that the protective role of Rhes may stem from its involvement in regulating autophagy through mTOR-independent pathways during the presymptomatic stage of HD.

Our results revealed that depletion of Rhes in HD KI mice via AAV transduction caused remarkable accumulation of mHTT at 22 weeks old (8 weeks after injection), but have no obvious effect at 36 weeks old HD KI mice (8 weeks after injection). Rhes has been reported to as a SUMO-E3 ligase and is capable of SUMOylating and stabilizing soluble mHTT in cell models (Subramaniam et al., 2009, 2010), it is plausible to suggest that the depletion of Rhes may result in reduced sumoylation of soluble mHTT, potentially promoting the aggregation of mHTT during the presymptomatic stage in HD KI mice. Furthermore, there was neurodegeneration (neuronal loss and astrogliosis) concurrent with the appearance of mHTT aggregations in the 22-week-old HD KI mice. These findings suggest that Rhes may play a protective role during the presymptomatic stage in HD KI mice. This is consistent with previous studies that showed Rhes deletion in BACHD transgenic mice enhanced striatal atrophy while overexpression of Rhes improved brain pathology in N171-82Q transgenic mice (Lee et al., 2014, 2015). It is possible that the function of autophagy remains unaffected during this stage, but when Rhes was deleted, it inhibits the autophagy activity, leading to aggregation of mHTT and exacerbation of neurodegeneration (neuronal loss and astrogliosis). However, during the symptomatic stage, the autophagy system in HD KI mice is already impaired (Brattas et al., 2021), and the deletion of Rhes might not worsen the situation.

Although we have strong evidence for the Rhes affecting mHTT aggregates formation in the early stage of HD KI mouse brain striatum, some limitations exist. Firstly, one of the limitations of this study is the relatively small sample size and future work with more animals is needed to enhance the robustness of our findings. Secondly, we only studied Rhes effects in one HD mouse model, which may limit the generalizability of our findings to other models or human patients. The use of different mouse models with varying genetic backgrounds, transgenic or knock-in models, and different HTT constructs (exon1 or full-length) may contribute to the discrepancies observed in previous studies regarding the role of Rhes in HD. Thirdly, we focused on investigating the effects of Rhes depletion at two specific time points, which may not fully capture the dynamic changes in disease progression. In addition, Rhes is recently reported to transport mHTT from striatum to other brain regions (Sharma and Subramaniam, 2019; Ramirez-Jarquin et al., 2022). It is important to consider the potential spatial-temporal variations in Rhes function and its impact on neurodegeneration in future studies. Existing evidence indicates that Rhes is a multifaceted player in HD pathogenesis, participating through sumoylation, mTOR-dependent, or mTOR-independent manner to regulate autophagy and other processes. Therefore, intervening with endogenous Rhes in HD models and systematically assessing its impact on various functions could potentially provide valuable insights into its role in the development of HD. Furthermore, the majority of current studies on Rhes in HD have been conducted using HD mouse models, it would be valuable to explore the role of Rhes in patient-derived HD cell models or large animal models to provide a more comprehensive understanding of its involvement in HD pathogenesis.

## Materials and methods

### Animals

All mice were bred and maintained in the animal facility in a 12:12 h light/dark room in accordance with the institutional guidelines of the Animal Care and Use Committee at Central South University. Full-length mutant HTT knock-in (140CAG) (HD KI) mice and Cas9 transgenic mice were obtained from Professor Xiao-Jiang Li at Jinan University. Mouse genotyping was performed by PCR using genomic DNA extracted from the mouse tails with primers, *HTT*, forward: 5′-ACT GCT AAG TGG CGC CGC GTA G-3′ and reverse: GAC GCA GCA GCG GCT GTG CCT G; Cas9, common forward: AAG GGA GCT GCA GTG GAG TA, WT reverse: TCG AAA ATC TGT GGG AAG TC, and transgenic reverse: TGG GCC ATT TAC CGT AAG TTA T.

### Plasmids and viruses

AAV-*Rasd2*-gRNAs were generated by inserting gRNA into a modified PX552 vector (Yang et al., 2017) via the *Sap*I restriction site. gRNA sequences are: gRNA-T1: CAA GGT GTA CAA CAT CCA CG ggg, gRNA-T2: TAA GAA CTC CTA CCG CAT GG tgg, gRNA-T3: CCA GCT GGT ACA TGT CCC CG tgg and gRNA-T4: CCA GTA CAC GCC CAC TAT CG agg, and control gRNA: ACC GGA AGA GCG ACC TCT TCT [PAM sequences are shown in lowercase; control gRNA was verified previously (Yang et al., 2017)]. All constructs were confirmed by double-direction Sanger sequencing. The plasmids were packaged into AAVs using serotype 9 capsids.

### Antibodies

Primary antibodies used in this study include Rhes (Genetex, GTX85428), mEM48 (QED Bioscience, 70102), NeuN (Cell Signaling Technology, 24307s), GFAP (Cell Signaling Technology, 3670), vinculin (Invitrogen, V9264), P62 (Abcam, ab56416), mTOR (Cell Signaling Technology, 2983), p-mTOR (Cell Signaling Technology, 5336), LC3 (Novus, NB100-2332), Beclin-1 (Cell Signaling Technology, 3495), p-Beclin-1 (Ser15, Cell Signaling Technology, 84966). Secondary antibodies were donkey anti-rabbit, and donkey anti-mouse Alexa Fluor 488 or 594 from Jackson ImmunoResearch.

### Stereotaxic injection

Surgical procedures were performed in accordance with the guidelines for the Care and Use of Laboratory Animals and biosafety procedures at Central South University. Stereotaxic injection of AAV into mouse brains was performed following the protocol described in our previous studies (Liu et al., 2020a). Briefly, mice were anesthetized with 1.5% isoflurane inhalation and stabilized on a stereotaxic instrument (David Kopf Instruments). Meloxicam was injected (5 mg/kg) as an analgesic. The injection site of the striatum was determined based on the distance from the bregma following coordinates (Yang et al., 2017): anterior-posterior = +0.55 mm, medial-lateral = ±2 mm, dorsal-ventral = −3.5 mm. Two microliters of AAV-gRNA virus (10^12^ genome copies/ml) per side was injected using a 30-guage Hamilton microsyringe at 200 nl/min speed. The microsyringe was left in the injection site for 10 min before it was withdrawn slowly. After the surgery, mice were placed on a heated blanket for recovery and monitored for 5 consecutive days.

### Cell culture

N2a cells were cultured in Dulbecco’s modified Eagle’s medium supplemented with 10% FBS, 100 μg/ml penicillin, and 100 units/ml streptomycin at 37°C with 5% CO_2_. Cells were transfected with 1 μg/well of DNA in a 12-well plate using lipofectamine 2000 (Invitrogen) for 48 h.

#### T7 endonuclease I assay

T7 endonuclease I assay was performed as described previously (Liu et al., 2020b). In brief, genomic DNA was isolated from Neuro2a cells transfected with a combination of Cas9 and gRNA plasmids. The target genomic region was amplified with the following primers: *Rasd2* T7 primers, forward: TGC CTT TCC CTC CTT CCT AT, and reverse: GAG AGA CCT ACC CTG GGG AC. The PCR products were reannealed under the following conditions: 95°C for 5 min, ramp to 4°C by 1°C/30 s, and then incubated with T7 Endonuclease I (New England Bio Labs) for 60 min at 37°C. The reaction products were subjected to 2% agarose gel electrophoresis.

### Immunohistochemistry

Mice were anesthetized and perfused intracardially with 0.9% saline solution, followed by 4% paraformaldehyde (PFA). Isolated mouse brains were post-fixed in 4% PFA overnight and then transferred into 30% sucrose for 48 h at 4°C. The completely dehydrated brains were sectioned at 30 μm by freezing microtome and stored in 0.1% NaN_3_ in phosphate-buffered saline.

For immunohistochemistry, brain sections were permeabilized with 0.3% Triton X-100/PBS at room temperature for 1 h and then treated with citric acid buffer at 95°C for 15 min for antigen retrieval. After being blocked with 4% normal goat serum in 0.3% Triton X-100/1X PBS for 1 h, brain sections were incubated with primary antibodies overnight at 4°C. After three rounds of 1X PBS wash, brain sections were incubated with secondary antibodies. Brain sections were developed with the Avidin-Biotin Complex Kit (Vector ABC Elite, Burlingame, CA, USA) and Sigma Fast DAB kit (Millipore Sigma).

### Western blot

Transfected N2a cells or AAV-injected striatum of HD mice were lysed in ice-cold RIPA buffer with freshly added protease inhibitor cocktail. Samples were sonicated and heated at 100°C for 10 min to denature the protein. Proteins were separated by SDS-PAGE electrophoresis and then transferred onto a nitrocellulose membrane. After being blocked with 5% milk in 1X PBS, the membrane was incubated with the primary antibody in 3% BSA/1X PBS at 4°C overnight. The membrane was then incubated with HRP-conjugated secondary antibodies in 5% milk/1X PBS for 1 h at room temperature and then developed with ECL Prime (GE Healthcare).

### Statistical analysis

The densitometric data (provided in Supplementary material) were obtained by the software ImageJ (1.51) and bar graphs were generated by the software GraphPad Prism (9.3.1). Two-tailed Student’s *t*-test was used to compare two groups. There were at least three injected mice striatum per group. All quantification data were presented as mean ± SEM. A *P*-value of less than 0.05 was considered statistically significant.

## Data availability statement

The original contributions presented in this study are included in the article/Supplementary material, further inquiries can be directed to the corresponding authors.

## Ethics statement

The animal study was approved by the Animal Care and Use Committee at Central South University. The study was conducted in accordance with the local legislation and institutional requirements.

## Author contributions

YP designed the study, performed most experiments, and wrote the manuscript. BT provided the advice, technical assistance, and support to the study. YP and QL collected and analyzed the data. X-JL, SL, and QL supervised the study and revised the manuscript. All authors contributed to the article and approved the submitted version.
